# Agrobacterium tumefaciens Growth Pole Ring Protein: C Terminus and Internal Apolipoprotein Homologous Domains Are Essential for Function and Subcellular Localization

**DOI:** 10.1128/mBio.00764-21

**Published:** 2021-05-18

**Authors:** John Zupan, Zisheng Guo, Trevor Biddle, Patricia Zambryski

**Affiliations:** a Department of Plant and Microbial Biology, University of California, Berkeley, Berkeley, California, USA; University of Massachusetts Amherst

**Keywords:** *Agrobacterium*, growth pole ring protein, apolipoprotein, bacterial polar growth, morphogenesis

## Abstract

The *Agrobacterium* growth pole ring (GPR) protein forms a hexameric ring at the growth pole (GP) that is essential for polar growth. GPR is large (2,115 amino acids) and contains 1,700 amino acids of continuous α-helices. To dissect potential GPR functional domains, we created deletions of regions with similarity to human apolipoprotein A-IV (396 amino acids), itself composed of α-helical domains. We also tested deletions of the GPR C terminus. Deletions were inducibly expressed as green fluorescent protein (GFP) fusion proteins and tested for merodiploid interference with wild-type (WT) GPR function, for partial function in cells lacking GPR, and for formation of paired fluorescent foci (indicative of hexameric rings) at the GP. Deletion of domains similar to human apolipoprotein A-IV in GPR caused defects in cell morphology when expressed in *trans* to WT GPR and provided only partial complementation to cells lacking GPR. *Agrobacterium*-specific domains A-IV-1 and A-IV-4 contain predicted coiled coil (CC) regions of 21 amino acids; deletion of CC regions produced severe defects in cell morphology in the interference assay. Mutants that produced the most severe effects on cell shape also failed to form paired polar foci. Modeling of A-IV-1 and A-IV-4 reveals significant similarity to the solved structure of human apolipoprotein A-IV. GPR C-terminal deletions profoundly blocked complementation. Finally, peptidoglycan (PG) synthesis is abnormally localized circumferentially in cells lacking GPR. The results support the hypothesis that GPR plays essential roles as an organizing center for membrane and PG synthesis during polar growth.

## INTRODUCTION

Polar growth is a mode of rod-shaped bacterial cell elongation in which new peptidoglycan (PG) is inserted specifically at one pole or at both poles in both Gram-positive and Gram-negative species (1–5). The order *Rhizobiales* (*Alphaproteobacteria*) is particularly rich in species that grow by polar growth, including the plant pathogen Agrobacterium., the nitrogen-fixing plant endosymbiont Sinorhizobium, and the animal pathogens Brucella and Bartonella (1, 5). It is notable that many pathogenic species exhibit polar growth, suggesting that pathways and mechanisms essential for polar growth may be targets for the development of new antibiotic strategies (6).

**FIG 1:**
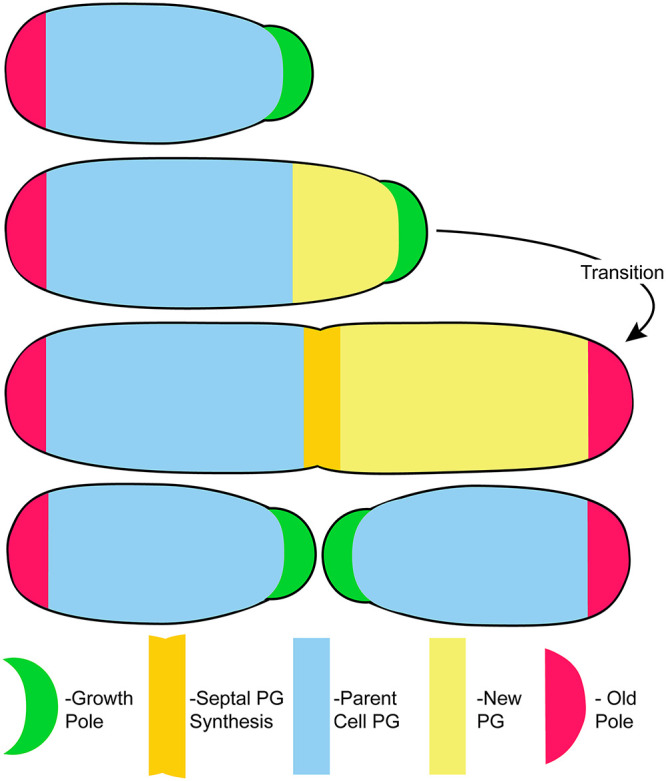
The essential features of polar growth in *Agrobacterium*. Cell elongation occurs from a single (GP) (green). When elongation is complete, the GP transitions into a nongrowing old pole (OP) (red). Peptidoglycan (PG) synthesis at the GP drives cell elongation (pale yellow), and PG synthesis is redirected to the septum (darker yellow) during cell division. After cell division, PG synthesis and polar growth resume in both sibling cells from the respective new GPs created by septation. PG synthesis does not occur in parental cells (blue). Adapted from Fig. 1 in Cameron et al. (5).

Agrobacterium tumefaciens has become a model system to investigate polar growth. Figure 1 summarizes the essential features of polar growth in *Agrobacterium*. First, growth occurs from a single growth pole (GP). Once new growth results in the length of a mature cell, the GP stops growing and transitions into a nongrowing old pole (OP). Following cell division, new GPs are formed at the septal sites of daughter cells (1, 3, 4, 7). Research to date has focused on proteins that localize to the growth pole (GP). The canonical cell division proteins FtsZ and FtsA localize to GP during polar growth and remain associated with the GP that forms at the site of septation in each sibling immediately after cell division (7). Although a GP function for FtsA has not been identified, FtsZ functions in the transition of a GP to an old pole (OP) (8). PodJ marks the OP throughout the cell cycle, but its gradual accumulation at the GP suggests it is also involved in the GP-OP transition (9, 10). PodJ is required for retention of the three largest (circular and linear chromosomes and megaplasmid) genetic elements of Agrobacterium tumefaciens at the OP to ensure faithful segregation of the genome to siblings (11). PopZ is a GP marker (10) and is required for the postreplication segregation of the three largest genetic elements to the Agrobacterium GP (11, 12). While PopZ and PodJ affect various aspects of polar growth and the cell cycle, polar growth still occurs in their absence (10–12).

Previously, we identified the Agrobacterium growth pole ring (GPR) protein as essential for polar growth (13). In its absence, cells lose the rod shape and adopt an essentially spherical morphology (13). Furthermore, very slight overexpression of GPR leads to the development of multiple growth poles (13). GPR is classified as an apolipoprotein (14, 15) and is predicted to be anchored in the bacterial membrane by two N-terminal transmembrane domains that position the bulk of the protein in the cytoplasm (13). GPR contains 2,115 amino acids with 1,700 amino acids of continuous α-helices containing multiple potential apolipoprotein-similar domains; in contrast, *bona fide* lipid transport apolipoproteins are single-domain small proteins of approximately 250 to 400 amino acids (14). Most remarkably, GPR localizes as six foci that form a 200-nm-diameter ring approximately 100 nm from the tip of the GP. These GPR characteristics, and the fact that it is essential for cell shape, led to the hypothesis that GPR functions as a scaffold for assembly of cellular growth machinery, especially enzymes involved in membrane and PG synthesis, to the GP (13).

To identify domains required for GPR function, we constructed internal and C-terminal GPR deletions fused to GFP coding sequences. Each GFP-GPR fusion protein was expressed both in *trans* to the WT protein and in the *gpr* depletion strain (13) (described below) and assayed for their effect on cell shape and polar localization. Deletion of GPR domains similar to human apolipoprotein A-IV caused defects in cell morphology when expressed in *trans* to wild-type (WT) GPR and provided different degrees of partial complementation to cells lacking GPR (relative to the complete complementation observed when GFP fused to full-length GPR is expressed). C-terminal deletions of 221 or 332 amino acids strongly blocked complementation. All deletion proteins were tested for their ability to form paired polar fluorescent foci, which are indicative of GPR hexamer formation. Notably, GPR deletions with the strongest alterations in cell morphology were unable to form paired polar foci, underscoring the importance of GPR structure for its function(s).

## RESULTS AND DISCUSSION

### Identification of domains for deletion in the GPR protein.

The bioinformatic strategy that identified the growth pole ring (GPR) protein and its unexpected similarity to apolipoproteins (Pfam PF01442) were previously described (13); at that time, prokaryote apolipoproteins represented 40% of the total known apolipoproteins. Due to the availability of more sequenced genomes and thus more proteome data, prokaryotic apolipoproteins currently represent 26% (293 proteins in 276 bacterial species versus 828 proteins [74%] in 178 eukaryote species; see Table 1 and Fig. S1 in the supplemental material). Notably, of the PF01442 proteins identified in bacteria, the majority (80%, 237/293) are found in the *Rhizobiales* (Table 1). This wide distribution in bacteria suggests that apolipoprotein homologous domains may have widespread function. Indeed, bacterial apolipoprotein homologous proteins are structural components of lipid droplets in several bacterial species (16–18), suggesting that lipid-binding function may be conserved in other bacterial proteins containing apolipoprotein domain(s).

**TABLE 1:** Distribution of PF01442 proteins among taxa^*a*^

Taxonomic group	No. of species with proteins in PF01442	No. of proteins in PF01442 (% of total)
All species	454	1,121 (100%)
Eukaryotes	178	828 (74%)
Bacteria	276	293 (26%)
*Rhizobiales*	225	237 (21%)
Others	51	56 (5%)

a Proteins in the Pfam apolipoprotein family (PF01442) are found in both eukaryotic and prokaryotic species (30). Among bacteria, these proteins are especially abundant in the *Rhizobiales*.

10.1128/mBio.00764-21.1 FIG S1 Species distribution of apolipoproteins in PF01442 (pfam.xfam.org/family/Apolipoprotein#tabview=tab7). Data mining of bacterial genomes and their proteomes reveals that ApoLPs are found in numerous bacterial species, especially those belonging to the order *Rhizobiales* (of which *Agrobacterium* is a member) of *Alphaproteobacteria*. As of 14 January 2021, prokaryotic ApoLPs represent approximately 26% of the total PF01442 family members (S. El-Gebali, J. Mistry, A. Bateman, S. R. Eddy, et al. Nucleic Acids Res 47:D427–D432, 2019, https://doi.org/10.1093/nar/gky995). See also Table 1. Download FIG S1, PDF file, 0.3 MB. Copyright © 2021 Zupan et al. 2021 Zupan et al. https://creativecommons.org/licenses/by/4.0/ This content is distributed under the terms of the Creative Commons Attribution 4.0 International license.

Pfam (19) identified multiple overlapping, almost continuous domains from the apolipoprotein family (PF01442) in GPR that cover approximately 75% (∼1,588 amino acids) of the protein (see Fig. S2 in the supplemental material). Furthermore, GPR-like secondary structures are conserved in the *Rhizobiales*, i.e., two N-terminal transmembrane domains and multiple domains similar to apolipoproteins (Pfam; see PF01442, domain organization). The widespread occurrence of the GPR architecture and demonstrated presence in lipid droplets is consistent with the hypothesis that GPR function involves lipid metabolism.

10.1128/mBio.00764-21.2 FIG S2 Pfam identified 10 domains in the apolipoprotein family (PF01442) in the growth pole ring (GPR) based on a hidden Markov model derived from 95 sequences. The position of each domain in the GPR is indicated by a black line. The exact amino acid limits of each domain are described in Supplemental Table 1 in J. R. Zupan, R. Grangeon, J. S. Robalino-Espinosa, N. Garnica, et al. (Proc Natl Acad Sci U S A 116:10962–10967, https://doi.org/10.1073/pnas.1905900116). Transmembrane domains are indicated in red. Download FIG S2, PDF file, 0.03 MB. Copyright © 2021 Zupan et al. 2021 Zupan et al. https://creativecommons.org/licenses/by/4.0/ This content is distributed under the terms of the Creative Commons Attribution 4.0 International license.

**FIG 2:**
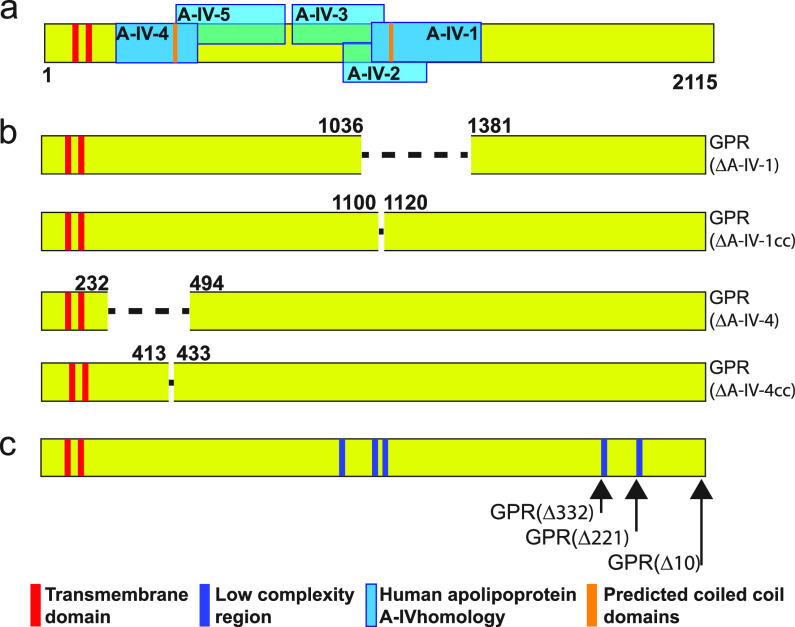
Growth pole ring protein bioinformatics and deletion strategy. (a) Location of GPR domains similar to human apolipoprotein A-IV (blue boxes). Boxes are numbered according to bit score (1 = highest, 5 = lowest; see Table S1 in the supplemental material). GPR is a protein of 2,115 amino acids. (b) Deletions of domains A-IV-1 and A-IV-4 and their coiled coil (cc) regions. Numbers indicate the beginning and end of the deleted amino acid sequence. Names of the constructs are on the right. (c) C-terminal deletions are indicated by arrows adjacent to their names. Numbers indicate the number of amino acids deleted from the C terminus. Low-complexity regions (23, 24) are defined in the text.

Thus, it is important to genetically test whether GPR apolipoprotein homologous domains play a role in GPR function. To assess the functional significance of GPR apolipoprotein domains, we first defined regions of GPR with the highest similarity to known apolipoproteins. We performed a UniProt-BLAST search with GPR of the human proteome (20), as human apolipoproteins are the best studied (14, 15), and identified apolipoprotein A-IV (GenPept accession number P06727), which aligned with five regions of GPR (Fig. 2a; see also Table S1 in the supplemental material). Regions of GPR with similarity to *bona fide* apolipoprotein domains are potentially functional domains. We selected two domains similar to apolipoprotein A-IV for deletion, GPR domain A-IV-1 because it had the highest bit score and percent amino acid identity to human apolipoprotein A-IV, and GPR domain A-IV-4 because it is proximal to the N terminus farthest from domain A-IV-1 (Fig. 2b; see also Fig. S3 and Table S1 in the supplemental material). GPR also contains potential coiled coil (CC) domains, which often mediate protein-protein interactions (21, 22), within domains A-IV-1 and A-IV-4 (Fig. 2a and Fig. S3); thus, we created deletions in the coding regions for each of these predicted CCs (Fig. 2b).

10.1128/mBio.00764-21.3 FIG S3 Amino acid sequences of GPR apolipoprotein domains analyzed here. Domains A-IV-4 (242 to 494) and A-IV-1 (1,036 to 1,381) are underlined. Amino acids shown in structural models in Fig. 6 (247 to 429 and 1,043 to 1,284) are in bold. Coiled coils (413 to 433 and 1,100 to 1,120) are in red. Download FIG S3, PDF file, 0.04 MB. Copyright © 2021 Zupan et al. 2021 Zupan et al. https://creativecommons.org/licenses/by/4.0/ This content is distributed under the terms of the Creative Commons Attribution 4.0 International license.

10.1128/mBio.00764-21.6 TABLE S1 GPR domains aligned to human apolipoprotein A-IV. Download Table S1, PDF file, 0.02 MB. Copyright © 2021 Zupan et al. 2021 Zupan et al. https://creativecommons.org/licenses/by/4.0/ This content is distributed under the terms of the Creative Commons Attribution 4.0 International license.

**FIG 3:**
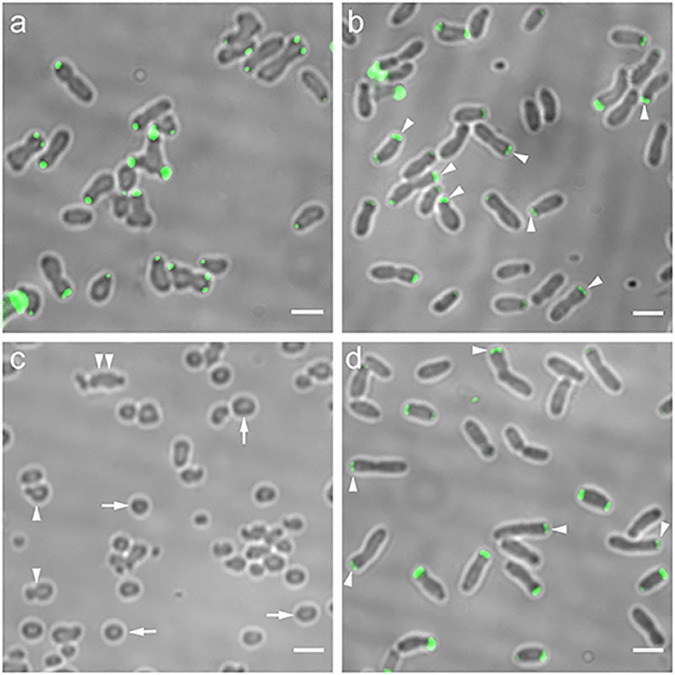
Cell shape phenotype and green fluorescent protein (GFP)-GPR localization by merodiploid interference (a and b) and complementation (c and d). (a) WT *Agrobacterium* expressing GPR-GFP (in *trans* to WT GPR) exhibits cell shape changes. (b) WT *Agrobacterium* expressing GFP-GPR (in *trans* to WT GPR) does not exhibit cell shape changes, and GFP-GPR localizes as paired foci (arrowheads). (c) *Agrobacterium* cells depleted of GPR are round (arrows), round with buds (arrowheads), or have multiple constrictions (double arrowheads). (d) Cell shape of *Agrobacterium* cells depleted of GPR is restored by expression of GFP-GPR, which localizes as paired foci (arrowheads). Bar, 2 μm.

Previous work suggested that the GPR C terminus plays a significant role in WT function, as expression of GFP fused to the C terminus of GPR in WT cells elicited numerous ectopic growth poles and an abnormally wide cell shape (Fig. 3a and reference 13). To test this hypothesis directly, we made genetic deletions at the 3′ end of the *gpr* coding sequence that resulted in the deletion of 10, 221, and 332 amino acids from the C terminus of GPR. This deletion strategy was guided in part by the bioinformatic identification of two low-complexity regions (LCRs) (Fig. 2c,) that may be important for structure and function (23, 24). LCRs have low amino acid diversity, and the frequency distribution of their amino acids deviates from what is commonly observed (23, 24). One or both C-terminal LCRs were deleted in GPR(Δ221) and GPR(Δ332), respectively.

All deletion constructs were designed so that deletion proteins were fused to GFP at their N termini, as GFP-GPR does not interfere with WT GPR function and complements a GPR depletion (Fig. 3d and reference 13).

### Strategy to assess functional domains of GPR using deletions of GPR.

We tested truncated forms of GPR for their ability to interfere with WT GPR function or to provide partial function when expressed in cells lacking GPR. First, interfering interactions of GPR deletions were assessed by expressing each GPR deleted protein as a fusion to the C terminus of GFP in WT cells. As a control, expression of GFP-GPR (full length) in *trans* to WT GPR does not affect cell shape (Fig. 3b) or localization as polar paired fluorescent foci (see Fig. S4a in the supplemental material and reference 13). Second, complementation (partial function) was assessed by expressing different deleted forms of GPR fused to GFP in cells depleted of GPR. As GPR forms a hexamer (13), mutant proteins may form functional homohexamers in the absence of WT protein (complementation assay) but nonfunctional mixed hexamers in the presence of WT protein (merodiploid assay). For these studies, we used a strain where the sequence of the native ribosome binding site at the *gpr* chromosomal locus was replaced by a theophylline-sensitive riboswitch sequence (25), so that *gpr* mRNA translation occurs only in the presence of theophylline. Thus, in the absence of theophylline, GPR is not produced, and cells display many abnormal shapes. Most cells are spherical; other phenotypes include “bowling-pin” shaped cells with a single bud, or cells with multiple constrictions (Fig. 3c and reference 13). All phenotypes are consistent with the lack of the GPR polar ring structure to act as an organizing center for localized polar growth. As a control for complementation experiments, Fig. 3d shows that in *trans* expression of full-length GFP-GPR restores WT cell shape and WT GPR localization as paired foci in the absence of theophylline-induced expression of chromosomal *gpr*.

10.1128/mBio.00764-21.4 FIG S4 Growth pole ring protein appears as paired foci in widefield fluorescence microscopy but is resolved into a ring of six foci in structured illumination microscopy (SIM). (a) The green fluorescent protein (GFP)-GPR fusion protein localize as paired foci in cells imaged by widefield fluorescence microscopy throughout the cell cycle (represented here by cells of increasing length [i, ii, iii, and iv, respectively]). (b) The GFP-GPR fusion protein appears as paired foci (i) or multiple foci (iii) when imaged by longitudinal SIM side views. The ring of GFP-GPR foci is revealed by rotating the three-dimensional reconstruction in the *z* axis of the SIM image (ii and iv, showing 6 or 5 visible foci). Bar, 500 nm. Download FIG S4, PDF file, 0.6 MB. Copyright © 2021 Zupan et al. 2021 Zupan et al. https://creativecommons.org/licenses/by/4.0/ This content is distributed under the terms of the Creative Commons Attribution 4.0 International license.

**FIG 4:**
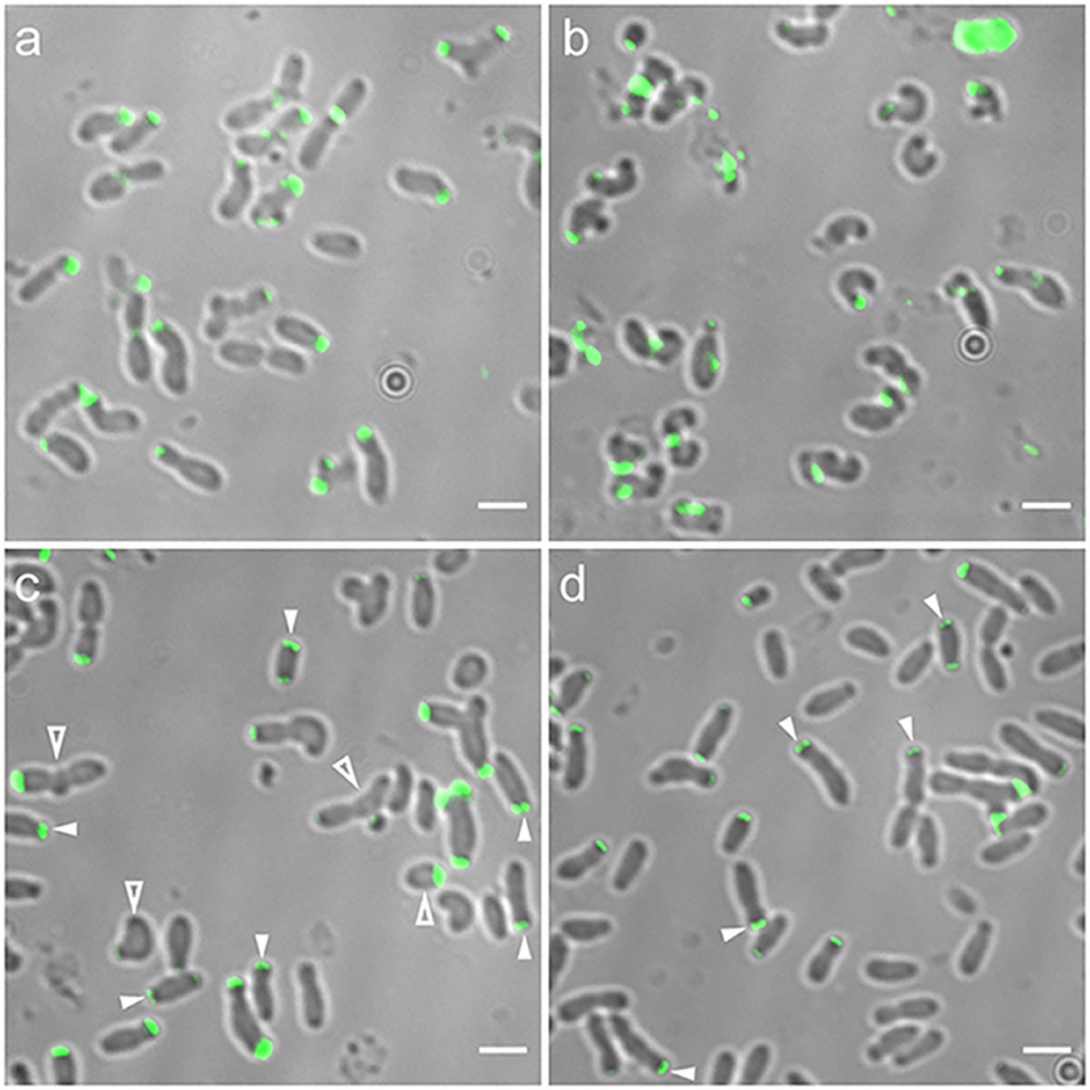
Cell shape and protein localization in cells expressing GFP-GPR(ΔA-IV-1) or GFP-GPR(ΔA-IV-1cc) assayed by merodiploid interference and complementation. (a) GFP-GPR(ΔA-IV-1) expressed in WT *Agrobacterium*. (b) GFP-GPR(ΔA-IV-1) expressed in GPR-depleted *Agrobacterium*. (c) GFP-GPR(ΔA-IV-1cc) expressed in WT *Agrobacterium.* (d) GFP-GPR(ΔA-IV-1cc) expressed in GPR-depleted *Agrobacterium*. Closed arrowheads, paired foci; open arrowheads, short cells or long cells with multiple constrictions. Bar, 2 μm.

**FIG 5:**
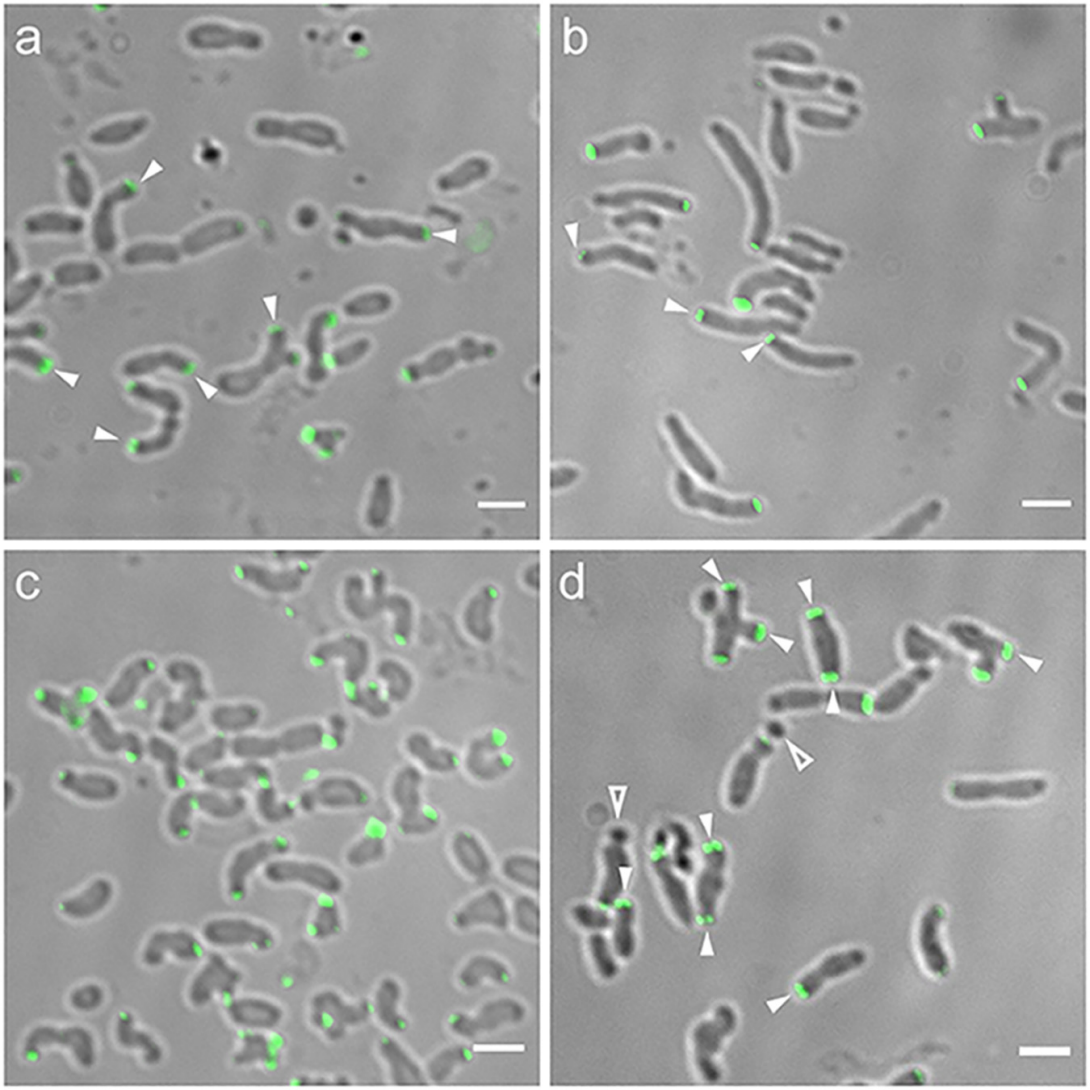
Cell shape and protein localization of cells expressing GFP-GPR(ΔA-IV-4) and GFP-GPR(ΔA-IV-4cc) assayed by merodiploid interference and complementation. (a) GFP-GPR(ΔA-IV-4) expressed in WT *Agrobacterium*. (b) GFP-GPR(ΔA-IV-4) expressed in GPR-depleted *Agrobacterium*. (c) GFP-GPR(ΔA-IV-4cc) expressed in WT *Agrobacterium.* (d) GFP-GPR(ΔA-IV-4cc) expressed in GPR-depleted *Agrobacterium*. Closed arrowheads, paired foci; open arrowhead, minicell. Bar, 2 μm.

**FIG 6:**
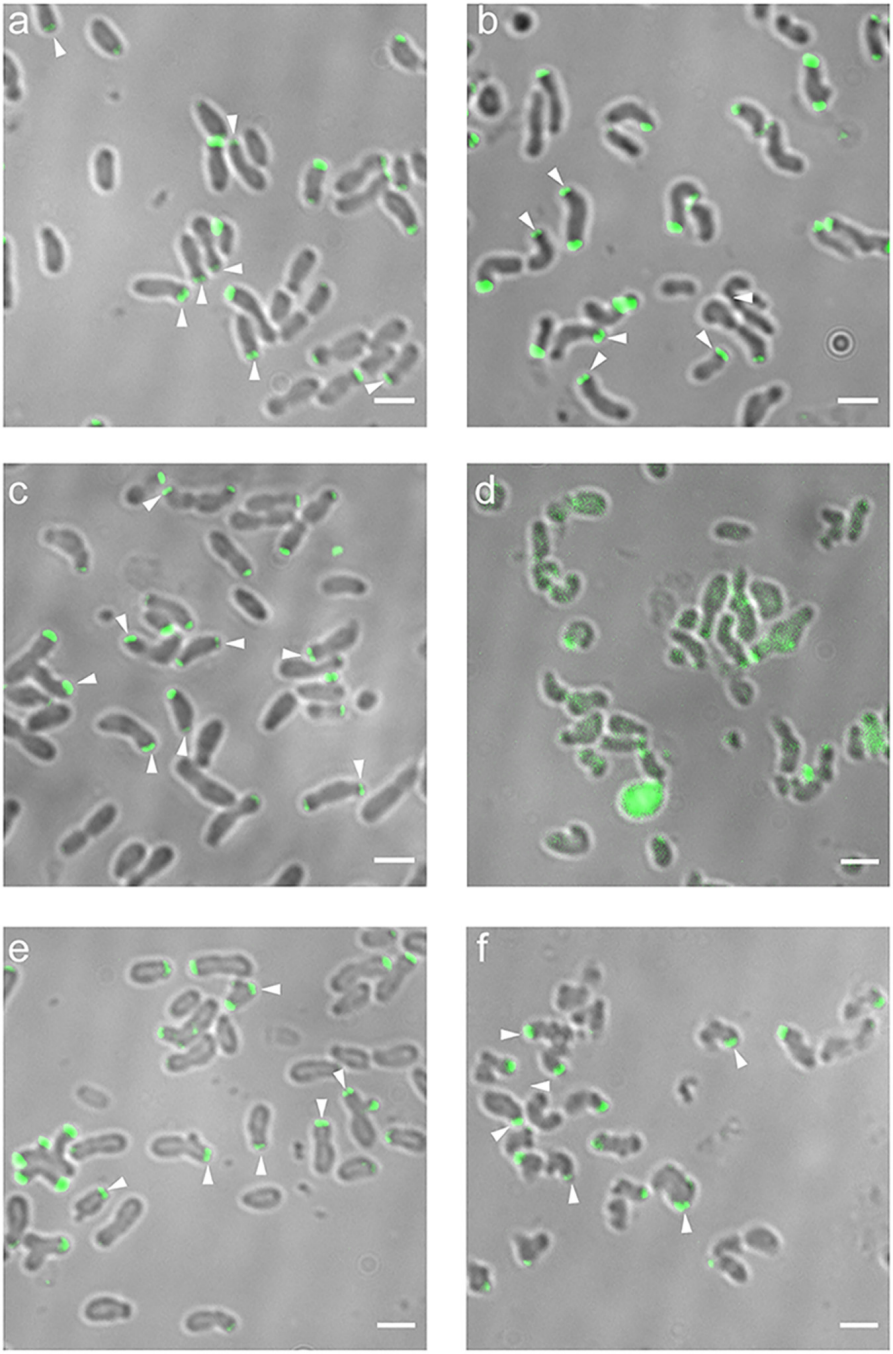
Cell shape and protein localization of cells expressing GFP-GPR(Δ10), GFP-GPR(Δ221), and GFP-GPR(Δ332), assayed by merodiploid interference and complementation. (a) Expression of GFP-GPR(Δ10) in WT *Agrobacterium*. (b) Expression of GFP-GPR(Δ10) in GPR-depleted *Agrobacterium*. (c) Expression of GFP-GPR(Δ221) in WT *Agrobacterium*. (d) Expression of GFP-GPR(Δ221) in GPR-depleted *Agrobacterium*. (e) Expression of GFP-GPR(Δ332) in WT *Agrobacterium*. (f) Expression of GFP-GPR(Δ332) in GPR-depleted *Agrobacterium*. Weak and few fluorescent foci were observed in cells with severe growth defects in panel d; such cells mostly exhibited weak, diffuse, nonlocalized fluorescent signal. Fluorescence in cells shown in panel d were visualized by increasing the fluorescent gain by 20%. Closed arrowheads, paired foci. Bar, 2 μm.

Here, we assess whether strains expressing different deletions of GPR interfere with WT GPR function and/or rescue the abnormal morphologies of cells lacking WT GPR. We monitor cell morphology as an indicator of loss or gain of GPR function. We assess localization of GFP-GPR fusions as paired fluorescent foci at the GP to assess WT localization (Fig. 3b and d). Such paired foci in single optical sections are indicative of the hexameric architecture of GFP-GPR observed by three-dimensional (3D) images obtained by structured illumination microscopy (SIM) (13) (Fig. S4b). Similar light exposure conditions were used to capture raw fluorescent images, and similar processing parameters were used to adjust the gain. Thus, the images of fluorescence foci in cells shown in Fig. 3 to 6 are directly comparable to each other. As in our previous publications (4, 7, 9–11, 13), we used low-copy-number plasmids and tightly controlled low-level expression lactose-inducible promoters so that GFP fusion proteins were not overproduced.

### Deletions of GPR apolipoprotein domain A-IV-1 and A-IV-1cc.

Cells expressing GFP-GPR(ΔA-IV-1) in *trans* to WT GPR exhibit significant alterations in cell shape (Fig. 4a). Although these cells retain GP-OP polarity, they also form ectopic GPs, and many cells are lumpy. The latter abnormal phenotype may be due the initiation of cell division constriction sites that do not complete septation (9, 26). GFP-GPR(ΔA-IV-1) localizes as single or broad foci at the GP. Thus, expressing a deletion of domain A-IV-1 interferes with WT GPR function and localization. As WT GPR protein is present in these cells, the abnormal phenotypes displayed following ectopic expression of GFP-GPR(ΔA-IV-1) imply that the deletion protein is interfering with WT GPR function, potentially due to formation of heterogeneous multimers composed of GPR and GFP-GPR(ΔA-IV-1). In GPR-depleted cells, GFP-GPR(ΔA-IV-1) exhibits very little function (Fig. 4b), as cells remain round or curved. Localization of GFP-GPR(ΔA-IV-1) is variable from polar to midcell, and no paired polar foci are observed. Thus, domain A-IV-1 is important for GPR function to promote polar growth and elongated cell shape, and loss of domain A-IV-1 interferes with both hexamer formation (merodiploid interference assay) and localization to the GP (complementation assay).

Cells expressing GPR(ΔA-IV-1cc) (20-amino-acid deletion of the predicted coiled coil in A-IV-1) in *trans* to WT GPR form ectopic poles, and are short, or elongated with multiple constrictions, similar to cells expressing GFP-GPR(ΔA-IV-1); however, GPR(ΔA-IV-1cc) forms paired foci, implying that loss of its CC-region does not interfere with hexamer formation (Fig. 4c). GPR(ΔA-IV-1cc) confers significant complementation when expressed in GPR-depleted cells, resulting in mostly normal elongated cells and paired polar foci, with few ectopic poles or lumpy cells (Fig. 4d).

### Deletions of GPR apolipoprotein domains A-IV-4 and A-IV-4cc.

GFP-GPR(ΔA-IV-4) elicits changes in cell shape both in *trans* to WT GPR and in complementation of GPR-depleted cells. GFP-GPR(ΔA-IV-4) interferes with WT GPR function, resulting in cells with variable diameter and length, ectopic GPs, and multiple constrictions (Fig. 5a). As suggested above, constrictions suggest Z rings may form but then abort. Nevertheless GFP-GPR(ΔA-IV-4) localizes to poles and forms paired foci. In GPR-depleted cells, GFP-GPR(ΔA-IV-4) results in partial complementation by producing rod-shaped cells with paired polar foci; however, most cells are abnormally long, with somewhat narrowed diameters (Fig. 5b). These latter phenotypes may reflect a requirement for domain A-IV-4 in GPR function during septation, as GPR localizes to the midcell just prior to cell division (Fig. S4a and reference 13).

Surprisingly, deletion of only 20 amino acids corresponding to the CC domain of A-IV-4 produces the most severe effects observed, as assessed by the merodiploid interference assay, for any of the deletion proteins analyzed here. When GFP-GPR(ΔA-IV-4cc) is expressed in *trans* to WT GPR, cells are short and very curved, and most cells show tight single foci (Fig. 5c). Short, rounded cells are reminiscent of GPR-depleted cells (Fig. 3c); thus, GFP-GPR(ΔA-IV-4cc) strongly interferes with WT GPR function. Furthermore, GFP-GPR(ΔA-IV-4cc) confers only partial complementation, resulting in longer cells with variable diameters and occasional ectopic poles and minicells (Fig. 5d). However, GFP-GPR(ΔA-IV-4cc) does form paired foci in cells lacking GPR (Fig. 5d).

The inability of GFP-GPR(ΔA-IV-4cc) to form paired foci when expressed in concert with the WT may reflect an inhibition of monomer-monomer interaction as a consequence of the severely curved shape of most cells that interferes with overall cell architecture at the GP. Or, GPR(ΔA-IV-4cc) may interfere more with GPR function when hexamer formation is derived from a mixed pool of GPR(ΔA-IV-4cc) deletion protein and WT protein monomers (merodiploid interference assay), but GPR(ΔA-IV-4cc) exhibits some function when assembled from a uniform pool of GPR(ΔA-IV-4cc) protein (complementation assay). Functional homomultimers and nonfunctional mixed multimers have been suggested to explain merodiploid interference versus complementation phenotypes for point mutants in the hexameric *Agrobacterium* VirB11 protein (27).

In summary, deletion of GPR apolipoprotein A-IV domains A-IV-1 or A-IV-4 causes severe effects on cell growth and morphology. However, only deletion of domain A-IV-1, not deletion of domain A-IV-4, interferes with formation of paired foci. Thus, domain A-IV-1 likely plays a significant role in the overall conformation of GPR monomers. Interestingly, deletion of the predicted CC domains in either A-IV-1 or A-IV-4 does not interfere with hexamer formation when either expressed on their own in the complementation assay, suggesting that monomer-monomer interaction does not require these CC regions. These data further imply that hexamer formation is not sufficient for function, as GPR(ΔA-IV-1cc), GFP-GPR(ΔA-IV-4), and GFP-GPR(ΔA-IV-4cc) form hexamers, but cells expressing these deletions are abnormally shaped. In the section “Structural predictions for domains A-IV-1 and A-IV-4,” we provide structural prediction data to suggest a rationale for the more severe effects of deletion of the CC in A-IV-4 on cell growth and morphology.

### Deletions at the C terminus of GPR.

GPR(Δ10) does not interfere with WT GPR function or localization (Fig. 6a; compare to expression of GFP-GPR in *trans* to WT GPR in Fig. 3b). However, in the complementation assay, GPR-depleted cells expressing GPR(Δ10) are abnormally curved, with multiple constrictions (Fig. 6b), suggesting that the C-terminal 10 amino acids are important for GPR function. GPR(Δ10) localizes as paired foci by both merodiploid interference or complementation assays, suggesting that the last 10 amino acids do not play a role in multimerization of GPR (Fig. 6a and b).

GPR(Δ221) also does not dramatically interfere with WT GPR function or localization, as the majority of cells are similar to WT in shape and display polar paired fluorescent foci; however, a few cells show abnormal shapes (Fig. 6c). Our assay is not quantitative *per se*, but it nevertheless presents evidence to suggest partial function or not. The results with GPR(Δ221) provide evidence that this deletion protein can interact with the WT GPR hexamer without inducing major defects. Unexpectedly, GPR(Δ221) exhibits the strongest inability to complement GPR-depleted cells observed here, as most GPR(Δ221) cells remain round and exhibit nonspecific diffuse GFP-GPR(Δ221) fluorescence (Fig. 6d). All other GPR deletion proteins expressed in the presence or absence of WT GPR (see above) exhibited some degree of foci formation either at the pole(s) or ectopically. Potentially, the severe loss of cell shape and underlying cell shape determinants induced by loss of the C-terminal 221 amino acids inhibits targeting of GFP-GPR(Δ221) to a discrete (polar) location. In support of this interpretation, when the normally tightly localized GP specific factor PopZ fused to GFP was expressed in round-shaped GPR-depleted cells, it exhibited diffuse localization (13). Thus, factors that determine GP function and shape may be unable to form and/or function in GPR(Δ221).

When GPR(Δ332) is expressed in *trans* to WT GPR, many cells are slightly broader in width and form branched ectopic poles at the poles. In GPR-depleted cells, GFP-GPR(Δ332) is unable to restore WT morphology and cell shape is very abnormal, with short and/or curved cells that sometimes contain multiple constrictions (Fig. 6f). Notably, the GPR(Δ332) deletion protein does not interfere with the formation of the GPR ring, indicated by the paired foci in either merodiploid interference or complementation assays. That GPR(Δ332) exhibits more function than GPR(Δ221) suggests the region between these two deletions has a negative influence on GPR function and targeting to the GP.

In summary, the C-terminal deletions suggest that the C terminus is essential for GPR function but is not essential for formation of the hexameric structure as paired foci form without the C-terminal 332 amino acids in both merodiploid interference and complementation assays. GPR(Δ221) provides several striking phenotypes. Loss of the C-terminal 221 amino acids produces a complete loss of GPR function, resulting in round cells and no polar foci (in the complementation assay); this severe phenotype compared to a longer C-terminal deletion in GPR(Δ332) suggests that the 111 amino acid region between amino acids 1,784 and 1,895 [present in GPR(Δ221)] interferes with structure and/or function of the GPR(Δ221) deletion protein. GPR(Δ332) lacks two LCRs (see Fig. 2c) (amino acids 1,787 to 1,800 and 1,895 to 1,926) while GPR(Δ221) still contains one LCR, which may cause its (more severe) phenotype. Both GPR(Δ221) and GPR(Δ332) lack a 110-amino-acid proline (P)-rich region (GPR amino acids 1,890 to 2,000), so its loss cannot explain their differing phenotypes. Notably, ectopic expression of WT GPR fused to GFP at its C terminus (GPR-GFP) blocks formation of paired foci (Fig. 3a), but loss of 332 amino acids at the C terminus does not; presumably the bulky GFP inhibits monomer-monomer interaction to form the GPR hexamer.

### Structural predictions for domains A-IV-1 and A-IV-4.

**FIG 7:**
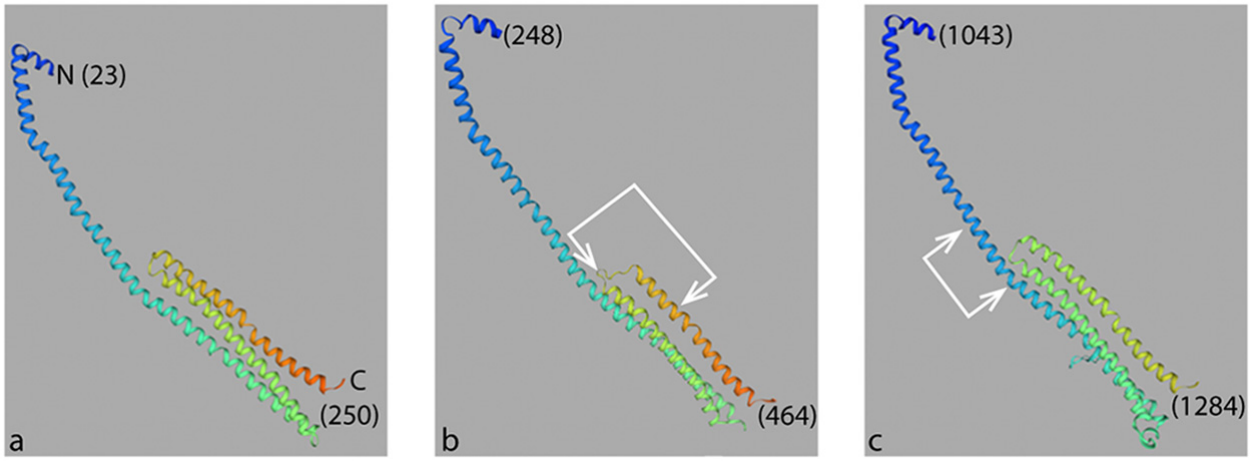
Structural models of domains GPR(A-IV-1) and GPR(A-IV-4). (a) Template 3s84.1 for human apolipoprotein A-IV consists of a long α-helix (blue) that is partially exposed and partially in a bundle with 2 additional α-helices (green and orange). N and C indicate the N terminus and C terminus, respectively, of the template structure. (b) Predicted structure of GPR domain A-IV-4. (c) Predicted structure of GPR domain A-IV-1. Predicted coiled coils deleted in GFP-GPR(ΔA-IV-1cc) and GFP-GPR(ΔA-IV-4cc) indicated by white arrows. Positions of these structural domains are given in Fig. S3 and Table S2 in the supplemental material. Numbers refer to amino acid positions in the three protein domains.

Previously, we suggested that GPR may exhibit 3D structural flexibility based on its classification in the Pfam apolipoprotein family PF01442 (13, 19). Since *Agrobacterium* polar growth requires extensive synthesis and remodeling of lipids at the GP, we proposed that GPR might form an organizing center for lipid (and peptidoglycan [PG]; see below) synthesis (13). Here, deletion of two specific regions of GPR with the strongest similarity to human apolipoprotein A-IV (Fig. 2a) exhibit significant defects in cell growth and morphology (Fig. 4 and 5), supporting the hypothesis that these *Agrobacterium*-specific apolipoprotein-similar regions play a role(s) in GPR function. To provide further support for this hypothesis, we investigated whether the 3D structures of GPR domain A-IV-4 (GPR amino acids 232 to 494) and GPR domain A-IV-1 (GPR amino acids 1,036 to 1,381) (Fig. 2a) resemble the well-characterized structure of human apolipoprotein A-IV (28, 29). The structural model for human apolipoprotein A-IV consists of a long exposed α-helix at the N terminus and a bundle of 3 α-helices at the C terminus (Fig. 7a).

10.1128/mBio.00764-21.7 TABLE S2 Amino acid positions of A-IV homology domains, SWISS-MODEL structural predictions, and predicted coiled coils (CC) within GPR. Download Table S2, PDF file, 0.01 MB. Copyright © 2021 Zupan et al. 2021 Zupan et al. https://creativecommons.org/licenses/by/4.0/ This content is distributed under the terms of the Creative Commons Attribution 4.0 International license.

SWISS-MODEL (30) predicts that both *Agrobacterium* GPR A-IV domains have striking structural similarities to human apolipoprotein A-IV. GPR amino acids 248 to 464 (within GPR domain A-IV-4, amino acids 232 to 494) and GPR amino acids 1,043 to 1,284 (within GPR domain A-IV-1, amino acids 1,036 to 1,381) form extended α-helices at their N termini and a bundle of 3 α-helices at their C termini, just like human apolipoprotein A-IV (Fig. 7a to c; see also Table S2 in the supplemental material). See Materials and Methods for details on structural modeling.

The structural models for GPR domains A-IV-1 and A-IV-4 may also explain the very strong merodiploid interference of GFP-GPR(ΔA-IV-4cc) relative to GFP-GPR(ΔA-IV-1cc). These small 20-amino-acid deletions (based on CC predictions) occur in different locations within the predicted α-helical regions. Amino acids 413 to 434 in GPR domain A-IV-4 span amino acids within the 3-helix bundle (Fig. 7b, arrows). In contrast, amino acids 1,100 to 1,120 in GPR domain A-IV-1 occur within the extended α−helix (Fig. 7c, arrows) without deleting amino acids in the other α-helices. Thus, deletion of amino acids 413 to 433 may more profoundly alter the 3D structure of the A-IV-4cc deletion protein. Indeed, GFP-GPR(ΔA-IV-4cc) cannot form paired foci in the merodiploid assay, while GFP-GPR(ΔA-IV-1cc) can.

However, this speculation does not explain why both GFP-GPR(ΔA-IV-4cc) and GFP-GPR(ΔA-IV-1cc) form paired foci when expressed alone in the complementation assay. Another possibility is that the CC of GPR A-IV-1 may not be needed for monomer-monomer interaction to form paired foci, but instead for interaction with another region of GPR or with a separate GP-specific factor. Indeed, the Sinorhizobium meliloti protein RgsE, a GPR homolog, may engage in multiple interactions with itself and other proteins involved in polar growth (31).

### Loss of GPR function alters localization of sites of peptidoglycan synthesis/remodeling.

**FIG 8:**
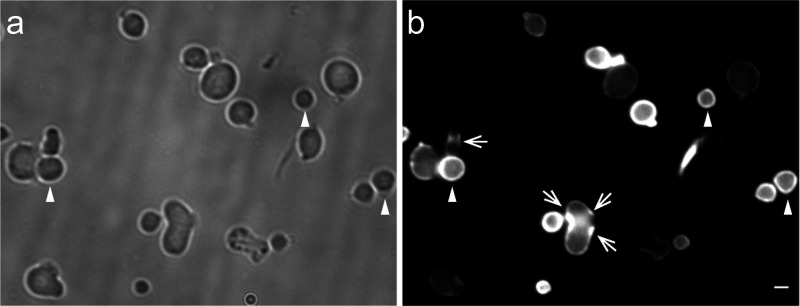
Peptidoglycan synthesis is not polarly localized in *Agrobacterium* Δ*gpr* cells. (a) Δ*gpr* cells are primarily spherical (arrowheads) and do not have an obvious growth pole. (b) PG synthesis (monitored by fluorescent PG precursors) is primarily distributed around the periphery of the cell (arrowheads). Arrows indicate regions with focused PG synthesis. Bar, 2 μm.

In *Agrobacterium*, exogenous alkyne-d-alanine (alkDala) is readily added to the terminal subunits of PG strands by l,d transpeptidase activity and can then be modified with an azido-fluorophore (see Materials and Methods), allowing simple and direct monitoring of the localization of PG synthesis/remodeling (1, 3, 4). Figure S5 in the supplemental material shows that WT *Agrobacterium* alkDala labeling occurs at the GP in both short cells during the growth phase early in the cell cycle and encompasses a larger area in longer cells late in the cell cycle. alkDala labeling then occurs at the midcell late, just before cell division (see also references 1 and 4). Thus, alkDala shows regions of the *Agrobacterium* cell in active or recent PG synthesis (1, 3, 4). To test the potential role of GPR in the spatial regulation and/or localization of PG synthesis/remodeling, we monitored the localization of alkDala in the *gpr* deletion strain that exhibits round-shaped cells. Without GPR, PG is distributed around the circumference of the cell (Fig. 8a and b) versus discrete locations during the polar growth cycle in WT *Agrobacterium* (1, 3, 4) (Fig. S5). Similarly, Mycobacterium smegmatis cells are rod shaped and grow by bipolar addition of PG (32, 33). M. smegmatis cells depleted of *divIVA*, however, are spherical, and PG synthesis is dispersed around their periphery (34). Some Δ*gpr* cells have regions of invagination that stain more intensely (Fig. 8b); such sites may represent PG synthesis during attempts at cell division, as we observed that even spherical Δ*gpr* cells can sometimes divide (13).

10.1128/mBio.00764-21.5 FIG S5 Peptidoglycan synthesis localization during the wild-type (WT) Agrobacterium tumefaciens cell cycle. Labelling of peptidoglycan synthesis is localized at the growth pole of small cells at the beginning of the cell cycle (single arrowheads). As the cells grow, labelling extends from the growth pole towards the midcell (double arrowheads). As the cell divides, peptidoglycan synthesis is labelled on both sides of the developing septation (arrows). Two fields of view are shown to present the range of variation in labelling. Bar, 2 μm. Download FIG S5, PDF file, 0.05 MB. Copyright © 2021 Zupan et al. 2021 Zupan et al. https://creativecommons.org/licenses/by/4.0/ This content is distributed under the terms of the Creative Commons Attribution 4.0 International license.

Thus, GPR is required to localize PG synthesis at the GP. Expression of GPR deletions either in *trans* to WT GPR or to complement GPR depletion produces a range of cell shapes from nearly WT to mostly spherical, suggesting specific GPR domains are essential to localize PG synthesis either directly or indirectly during polar growth.

### Conclusions.

When fused to GFP and viewed by 3D resolution microscopy, GPR forms a 200-nm-diameter fluorescent ring with six equally spaced 50-nm fluorescent foci (13). Here, we monitored the formation of adjacent paired fluorescent foci to assay the formation of GPR hexamers. The critical importance of this ring structure in GPR function is underscored by two specific GPR deletions (deletion of apolipoprotein domain A-IV-1 and deletion of the C-terminal 221 amino acids) that cannot form paired foci and exhibit the most defective cell morphology phenotypes in the complementation assay.

The predicted secondary structure of GPR, which consists predominantly of α-helices, does not offer easy clues into its structure or function. We identified five GPR domains (each approximately 260 to 350 amino acids) with similarity to human apolipoprotein A-IV. Deletion of two of these domains cause defects in cell morphology and growth. Besides amino acid sequence similarity, these two domains exhibit strong structural similarity to human apolipoprotein A-IV. How might these domains of GPR function? It is intriguing to consider that GPR, like apolipoproteins, is essential to sort and/or sequester lipids. Human apolipoproteins are small α-helical proteins that alter their conformation and dimerize for lipid sequestration (28, 35). For GPR to act in an analogous way, it must fold upon its very long length (2,115 amino acids) to potentially allow interaction between its individual apolipoprotein domains, as proposed previously (13). There are extensive possible folding conformations, given the length of GPR, and it is evident that folding must occur, as GPR foci are only 50 nm in diameter, whereas an extended conformation of its 1,700-amino-acid α-helical domain would be 600 nm (13).

Polar growth requires both membrane and PG synthesis. The large ring structure of GPR and its polar localization suggested that GPR might act as a scaffold to facilitate the organization of essential GP-specific proteins, such as those required for membrane and PG synthesis (13). The present results support this hypothesis because (i) GPR contains regions with homology to apolipoprotein domains that have well-established roles in lipid sorting (14, 15), and (ii) loss of GPR severely mislocalizes PG synthesis to the side walls of cells that lose their rod shape. Exactly how GPR might perform such functions is unknown, and provokes questions for future investigation. Is GPR localization dependent on interaction with lipids (or with PG)? Is the composition of membranes (or PG) at the growth pole distinct from the side walls of the bacterial cell? Does interaction of specific lipids (or PG) with GPR establish a unique GP-specific membrane (or PG) environment that recruits additional polar growth factors? Does the extensive α-helical character of GPR mediate protein-protein interactions at the growing pole?

## MATERIALS AND METHODS

### Bioinformatics.

The bioinformatic strategy that identified GPR was previously described (13). For this work, we then used the *Agrobacterium* GPR protein (Atu1348; NCBI reference sequence WP_010971556.1, UniProt identifier A9CJ72) as the query in a UniProt BLAST search of the Human Proteome (UniProt identifier UP000005640) with default parameters (20). From this search, we identified five regions of GPR protein with similarity to human apolipoprotein A-IV (Fig. 2a and Table S1 in the supplemental material. We also used Bioinformatics Toolkits PCOILS (36) (14-amino-acid window) to predict two 21-amino-acid coiled coils in human apolipoprotein A-IV-similar domains A-IV-1 and A-IV-4 (Fig. 2a and Fig. S3 in the supplemental material).

### Structural predictions.

For structural predictions, we submitted amino acid sequences for the A-IV-1 (amino acids 232 to 494) and A-IV-4 (amino acids 1,036 to 1,381) domains of GPR to SWISS-MODEL (30) and searched the database in the automated mode for templates. Models for these two domains were then built from the template with the best fit; for both domains, the best fit template was 3s84.1 for human apolipoprotein A-IV (Fig. 7a).

### Plasmid construction.

Standard molecular cloning techniques were used. All plasmids were derived from pJZ253 (*Plac*::*gfp-gpr* in pSRKKm [13]) by inverse PCR with phosphorylated primers (37). PCR was performed with the proofreading enzyme Phusion high-fidelity (HF) DNA polymerase and GC buffer (New England Biolabs). PCR conditions were determined empirically for each primer pair. Briefly, forward and reverse primers (20 to 25 mers) were designed to flank the coding sequence for the amino acids to be deleted. Primer sequences were derived from the nucleic acid sequence for *gpr* (*atu1348*; GenBank accession number AAK87140) and pSRKKm (38). Each PCR product was then ligated to generate the desired deletion in *gfp-gpr*. The original stop codon in pJZ253 was retained in all constructs. All constructs were confirmed by DNA sequencing to be in frame with the precise deletion. Resulting plasmids, based on pSRK vectors, placed cloned genes under the control of a tightly regulated lactose-inducible promoter, resulting in expression between 10 and 20% of WT levels (7). Table S3 in the supplemental material contains additional information on plasmids and strains.

10.1128/mBio.00764-21.8 TABLE S3 Bacterial strains and plasmids used in this study. Download Table S3, PDF file, 0.04 MB. Copyright © 2021 Zupan et al. 2021 Zupan et al. https://creativecommons.org/licenses/by/4.0/ This content is distributed under the terms of the Creative Commons Attribution 4.0 International license.

### Bacterial strains and growth conditions.

Cloning of the above-mentioned plasmids was performed using Escherichia coli XL Blue. All *Agrobacterium* strains were grown in Luria broth (LB) at 28°C. When appropriate, growth medium was supplemented with 40 μg/ml kanamycin to select for plasmids carrying GFP fusion constructs. The riboswitch-*gpr* strain was grown with or without 0.5 mM theophylline to either express GPR or deplete cells of GPR protein, respectively (13).

### Fluorescence and SIM.

Lactose-inducible expression of cloned genes was achieved by diluting overnight cultures to 10^8^ cells/ml and adding 0.25 mM isopropyl-β-d-thiogalactopyranoside (IPTG) for 10 h before widefield fluorescence or structured illumination microscopy (SIM) imaging as previously described (13). All images were processed using Fiji/ImageJ software (39). Similar levels of protein expression in A. tumefaciens are suggested for all constructs for the following reasons. First, the low level of expression from pSRKKm is described above. Second, similar light exposure conditions were used to capture raw fluorescent images for all constructs. Finally, similar processing parameters were used to similarly adjust the gain for all images using Fiji/ImageJ.

### Fluorescence labeling of peptidoglycan synthesis sites.

Alkyne-d-alanine labeling was conducted essentially as previously described (4). The only change in this protocol was to allow incorporation of (R)-α-propylargylglycine (Fisher Scientific) for 10 min, which optimized labeling for this strain.
