# Supplementary material for: Agrobacterium tumefaciens Growth Pole Ring Protein: C Terminus and Internal Apolipoprotein Homologous Domains Are Essential for Function and Subcellular Localization
Source: mBio. 2021 May 18;12(3):e00764-21. doi: 10.1128/mBio.00764-21 (PMC8262873; doi:10.1128/mBio.00764-21)
Supplement: FIG S1 [file mbio.00764-21-sf001.pdf]

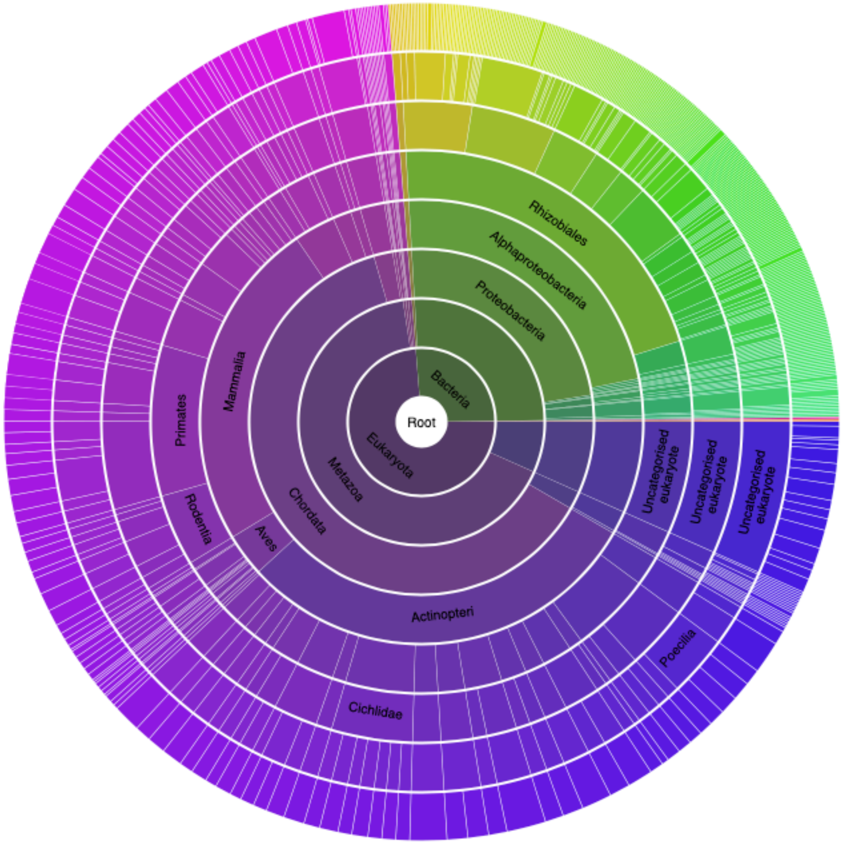

Figure S1. Species distribution of apolipoproteins in PF01442 (pfam.xfam.org/family/Apolipoprotein#tabview=tab7). Data mining of bacterial genomes and their proteomes reveals that ApoLPs are found in numerous bacterial species, especially the Rhizobiales order (where Agrobacterium is a member) of Alphaproteobacteria. As of January 14, 2021, prokaryotic ApoLPs represent approximately 26% of the total PF01442 family members (1). See also Table 1.

Reference

1. El-Gebali S, Mistry J, Bateman A, Eddy SR, Luciani A, Potter SC, Qureshi M, Richardson LJ, Salazar GA, Smart A, Sonnhammer ELL, Hirsh L, Paladin L, Piovesan D, Tosatto SCE, Finn RD. 2019. The Pfam protein families database in 2019. Nucleic Acids Res **47**:D427–D432.
