# Supplementary material for: Agrobacterium tumefaciens Growth Pole Ring Protein: C Terminus and Internal Apolipoprotein Homologous Domains Are Essential for Function and Subcellular Localization
Source: mBio. 2021 May 18;12(3):e00764-21. doi: 10.1128/mBio.00764-21 (PMC8262873; doi:10.1128/mBio.00764-21)
Supplement: FIG S2 [file mbio.00764-21-sf002.pdf]

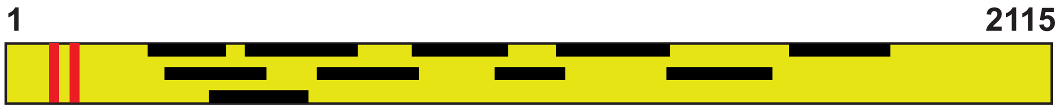

Figure S2. Pfam identified 10 domains in the apolipoprotein family (PF01442) in GPR based on a Hidden Markov Model derived from 95 sequences. The position of each domain in GPR is indicated by a black line. The exact amino acid limits of each domain are described in Supp. Table 1 in (1) . Transmembrane domains indicated in red.

Reference

1. Zupan JR, Grangeon R, Robalino-Espinosa JS, Garnica N, Zambryski P. 2019. GROWTH POLE RING protein forms a 200-nm-diameter ring structure essential for polar growth and rod shape in Agrobacterium tumefaciens. Proceedings of the National Academy of Sciences of the United States of America 116:10962–10967.
