# Supplementary material for: Agrobacterium tumefaciens Growth Pole Ring Protein: C Terminus and Internal Apolipoprotein Homologous Domains Are Essential for Function and Subcellular Localization
Source: mBio. 2021 May 18;12(3):e00764-21. doi: 10.1128/mBio.00764-21 (PMC8262873; doi:10.1128/mBio.00764-21)
Supplement: FIG S3 [file mbio.00764-21-sf003.pdf]

MANNKISDSVDETAFQALEDALQLGAFEEKPETRKTAKPKQPEARVKEASRQTPAPEATRAPVEQTPRSP
NLEAANDGSKRSPAMILKSLEGGSIGGALRNATIMSVIWALGGLGIAHLLYGNALWSIGSLADLTAIPGL
MAIVVGILVPVMLFFAFAIMMARARDLRNAARSMAEVALRLAEPETVASDRIMSVGQAVRREVSAMNDGI
ERTIARATELETLVHSEVNALERSYADNELRVRSLVQ**ELTAERDAIVNHAERIRSSIVGAQEQIKEELSI**
VGEELSMRIATTGEAFASMIDTRSAALLEKSRASTEAMGSLIAAKTENLLQALNSSGSTISNEFDMRLHN
LTSTLDERGEVLLERFAIHASTLDSGVESLNSALEERTRQLNETLSARSLELNRNIERGQQVIGGSLDTV
LDKLSTTLEEKGLSFRQSLQSTADDAIMDLDLRSGLYEERMQATVGQVNSAFDEHVAQFASAFDQRAGSL
DSKLMESLARINETVAGGSEALDTILTSGLERIGSTMTDQSLALATALGTGQEMLENALESRTQAFSDAI
GQRTAEITDAFTNSHAKIDTVLAERSNALFGALSASQDRFDEALASRSLAITGSVSGTAEHLAAMLDERA
AAINSVVADVERRLTETLETRAAAITGAVSGIEDRISDTLESRTAALHDVVSGAESRIADTLDGRTAALS
SAISGVEERIADTMDSRTLSLDMTFANVEERLSETLDNRTSALTGIVASAEEKIAGALDSRTATFGDVVA
GAETRIAETLDGRTAALNAVVSGAEERIADALDSRTMALDMTFSGAEEKIAEALDTRTAALGELVASAET
RIAGALDSRTDSLKTVVSGAEERITDVLDSRTMALDMSFSGVEEKITDILDGRTAALKSAVAGVEDRIAG
ALDSRTAALSGIVSGAEERIAEALDSRTLALDMTISGVEERIAEAMDARASSLSLAAAGVGQRLEATAFT
LENALASGHERLETMLGSQAERIAGSLERNSGLIEQSVSGAANRIENVVEDGSSRFAQTVEE**GVSRLENN**
LSQSHEEIRTALDQRQADLAATLSSATTQMGDMLSEQAMMIGTTVASSASMLELSLETQQDTLQKAIDGS
AATLEARLRNSAGDIAVKIGEAAREIGGATDALSTRIETSIGNVTTRLDETGARIETSLDALQTRVGGDL
ANVNNSIEDAGRRFADALEDKTAVFARTSDEAAERITGILDEQTTRVADTFENRTSRLAETFDAGTARID
ERLGTMDRALTIGLENVNRTIEGKASDLAVSLRGAVVSATQNIGDEAARSSALLAKSGSEFAEQVQAQNE
AFTKAIEERSGEIVTRISDAQTRLSGQAAAVAQTFSEAGNIIVNKVAEAEAVVRSQVGVISETLTSVESA
LDARGESIRSALDNRTRELNSMLASRSAELSRLIEEKAKPVVEEYATIGREAAEKIVSAAQQSAELLSQS
NSGMVGMVEQAISDYAAAGTDAASKLVAATRQSTDMLSETHTAMADAVEQGIDKYARSGSDAANKLVAAT
RQSTDMLSQTHNSMAEIVEQSATNFNAAVERAAQGFGAADEALNASATRFSESASQAADMVSSSSRLLEG
KIDRLSNISGQTLAQVAGIVGRFEEHSKVLSQASELLNAAQSSLVGTLEERQDALRSLSVGLVKRSEEIE
TAMRNVVGVVENTLNEAEERSQNVAGNLRDNLQASFSDIGRSLDETEQRARSAAQTMRGALLSAGQDASR
SIESTLSDAQKYSDELVNRLRGGVESSLSEVDNLLGSASEKSNAAAANLKETLRQAVEEAVSRFAGATDE
IRRSSHDIRRELDATRAELKRGAFDLPEEAKESAAAMRRAVSEQIKALQDISQLVGRSTHQMEVSEPVAR
AIAATQPAAERRVEPRPQPAAAAPVQQRPAQPQPAPALRGTLPLENRQVENRQAPAPAQPVATNPAGRRE
EGGGWISDLLRGASQETPAASTPRASTEQQPTRAADTRNPRHMVESLNSLSVDIARAIDHDASVELWRRY
QRGERDVFTRRLYTLKGQTTFDEIKRKYEREAEFRTAVDRYITDFEKLLADVARTDRDRSVTQSYLTSDT
GKVYTMLAHAAGRFN

Figure S3. Amino acid sequences of GPR apolipoprotein domains analyzed herein. Domains A-IV-4 (242-494) and A-IV-1 (1036-1381) are underlined. Amino acids shown in structural models in Figure 6 (247-429 and 1043-1284) are in bold. Coiled coils (413-433 and 1100-1120) are in red.
