# Supplementary material for: Agrobacterium tumefaciens Growth Pole Ring Protein: C Terminus and Internal Apolipoprotein Homologous Domains Are Essential for Function and Subcellular Localization
Source: mBio. 2021 May 18;12(3):e00764-21. doi: 10.1128/mBio.00764-21 (PMC8262873; doi:10.1128/mBio.00764-21)
Supplement: TABLE S1 [file mbio.00764-21-st001.pdf]

Table S1. GPR domains aligned to human apolipoprotein A-IV.

GPR homology domain	Position (amino acids)	Bit Score (E-value)	% Identity	Coiled Coil Position (amino acids)
A-IV-1	1036-1381	144 ($3.6x10^{-8}$)	19.9	1100-1120
A-IV-2	818-1115	112 ($3.6x10^{-8}$)	16.4	
A-IV-3	948-1211	104 ($3.6x10^{-8}$)	16.4	
A-IV-4	232-494	96 ($3.6x10^{-8}$)	18.1	413-433
A-IV-5	412-765	83 ($3.6x10^{-8}$)	17.8	
