# Supplementary material for: Agrobacterium tumefaciens Growth Pole Ring Protein: C Terminus and Internal Apolipoprotein Homologous Domains Are Essential for Function and Subcellular Localization
Source: mBio. 2021 May 18;12(3):e00764-21. doi: 10.1128/mBio.00764-21 (PMC8262873; doi:10.1128/mBio.00764-21)
Supplement: FIG S4 [file mbio.00764-21-sf004.pdf]

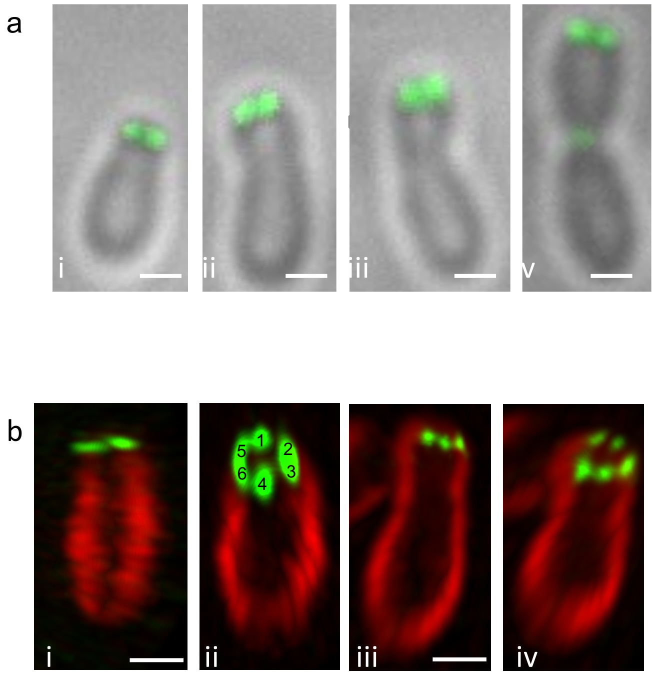

Figure S4. Growth pole ring protein appears as paired foci in widefield fluorescence microscopy but is resolved into a ring of six foci in structured illumination microscopy (SIM). a) GFP-GPR protein localizes as paired foci in cells imaged by widefield fluorescence microscopy throughout the cell cycle (represented here by cells of increasing length (i, ii, iii, and iv, respectively). b) GFP-GPR protein appears as paired foci (i) or multiple foci (iii) when imaged by longitudinal SIM side views. The ring of GFP-GPR foci is revealed by rotating the three-dimensional reconstruction in the Z-axis of the SIM image (ii and iv, showing 6 or 5 visible foci). Scale bar, 500nm.
