# Supplementary material for: Agrobacterium tumefaciens Growth Pole Ring Protein: C Terminus and Internal Apolipoprotein Homologous Domains Are Essential for Function and Subcellular Localization
Source: mBio. 2021 May 18;12(3):e00764-21. doi: 10.1128/mBio.00764-21 (PMC8262873; doi:10.1128/mBio.00764-21)
Supplement: TABLE S2 [file mbio.00764-21-st002.pdf]

Table S2. Amino acid positions of A-IV homology domains, Swiss Mode structural predictions, and predicted coiled coils (CC) within GPR.

GPR Domain	Position of A-IV homology domain (amino acid #)	Structural model position within A-IV homology domain (amino acid #)	Position of CC within A-IV homology domain (amino acid #)
A-IV-4	232-494	248-464	413-433
A-IV-1	1036-1381	1043-1284	1100-1120
