# Supplementary material for: Agrobacterium tumefaciens Growth Pole Ring Protein: C Terminus and Internal Apolipoprotein Homologous Domains Are Essential for Function and Subcellular Localization
Source: mBio. 2021 May 18;12(3):e00764-21. doi: 10.1128/mBio.00764-21 (PMC8262873; doi:10.1128/mBio.00764-21)
Supplement: FIG S5 [file mbio.00764-21-sf005.pdf]

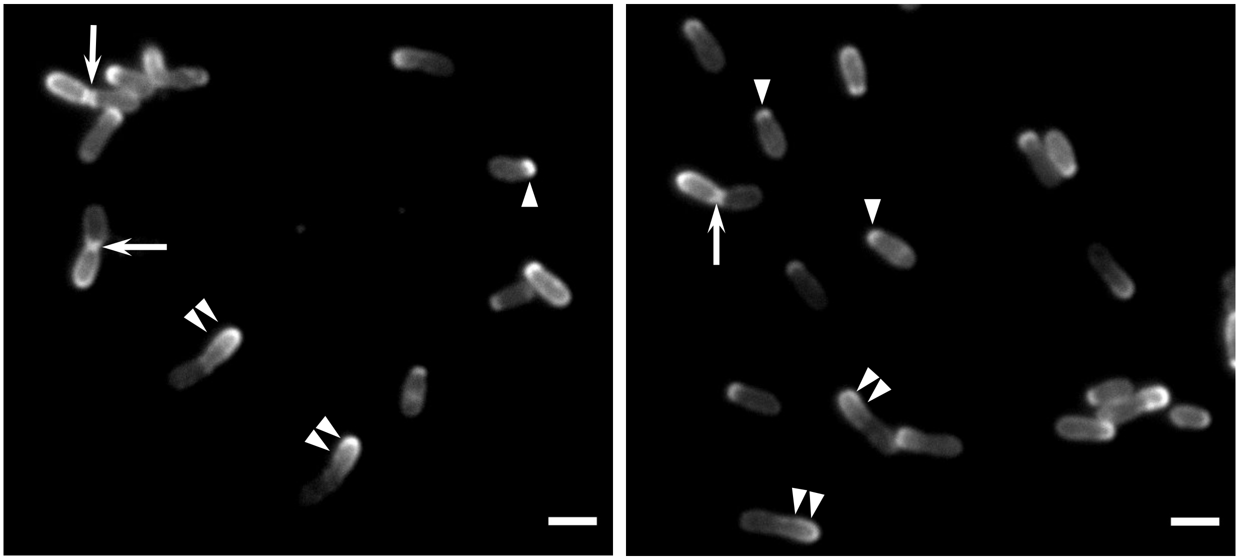

Figure S5. Peptidoglycan synthesis localization during the WT *Agrobacterium tumefaciens* cell cycle. Labelling of peptidoglycan synthesis is localized at the growth pole of small cells at the beginning of the cell cycle (single arrowheads). As the cells grow, labelling extends from the growth pole towards the midcell (double arrowheads). As the cell divides, peptidoglycan synthesis is labelled on both sides of the developing septation (arrows). Two fields of view are shown to present the range of variation in labelling. Scale bar, 2 μm.
