# Supplementary material for: Agrobacterium tumefaciens Growth Pole Ring Protein: C Terminus and Internal Apolipoprotein Homologous Domains Are Essential for Function and Subcellular Localization
Source: mBio. 2021 May 18;12(3):e00764-21. doi: 10.1128/mBio.00764-21 (PMC8262873; doi:10.1128/mBio.00764-21)
Supplement: TABLE S3 [file mbio.00764-21-st003.pdf]

Table S3: Bacterial strains and plasmids used in this study.

Plasmid	Description	Source
pSRKKm	Broad host-range, *lacI*, kanamycin^R	(1)
pJZ210	*Plac::eGFP,* gentamycin^R	(2)
pJZ251	*Plac::gpr-eGFP*, gentamycin^R<br>*gpr*= Atu1348, GenBank AA87140	(2)
pJZ253	*Plac::eGFP-gpr*, gentamycin^R<br>*gpr*= Atu1348, GenBank AA87140	(2)
pJZ281	*Plac::eGFP-gpr (∆332)*, gentamycin^R	This work
pJZ283	*Plac::eGFP-gpr (∆A-IV-1)*, gentamycin^R	This work
pJZ285	*Plac::eGFP-gpr (∆10)*, gentamycin^R	This work
pJZ287	*Plac::eGFP-gpr (∆221)*, gentamycin^R	This work
pJZ289	*Plac::eGFP-gpr (∆A-IV-4cc)*, gentamycin^R	This work
pJZ291	*Plac::eGFP-gpr (∆A-IV-1cc)*, gentamycin^R	This work
pJZ292	*Plac::eGFP-gpr (∆A-IV-4),* gentamycin^R	This work
**Strains**	**Relevant genotype**	**Source**
XL Blue<br>(*E. coli*)	cloning strain, endA1 gyrA96(nalR) thi-1 recA1 relA1 lac glnV44 F'[::Tn10 proAB+ lacIq Δ(lacZ)M15] hsdR17(rKmK+), TetR	Lab stock
C58	wild-type *A. tumefaciens* strain C58	Lab Stock
A185	C58 carrying pJZ251	(2)
A187	C58 carrying pJZ253	(2)
A217	C58 carrying pJZ281	This work
A219	C58 carrying pJZ283	This work
A221	C58 carrying pJZ285	This work
A223	C58 carrying pJZ287	This work
A224	C58 carrying pJZ289	This work
A237	C58 carrying pJZ291	This work
A240	C58 carrying pJZ292	This work

A212	*Riboswitch::gpr*	(2)
A231	A212 carrying pJZ289	This work
A233	A212 carrying pJZ283	This work
A235	A212 carrying pJZ281	This work
A236	A212 carrying pJZ285	This work
A238	A212 carrying pJZ291	This work
A239	A212 carrying pJZ253	(2)
A271	A212 carrying pJZ292	This work

References

1. Khan SR, Gaines J, Roop RM, Farrand SK. 2008. Bdroad-host-range expression vectors with tightly regulated promoters and their use to examine the influence of TraR and TraM expression on Ti plasmid quorum sensing. Applied and Environmental Microbiology 74:5053–5062.

2. Zupan JR, Grangeon R, Robalino-Espinosa JS, Garnica N, Zambryski P. 2019. GROWTH POLE RING protein forms a 200-nm-diameter ring structure essential for polar growth and rod shape in Agrobacterium tumefaciens. Proc Natl Acad Sci USA 116:10962–10967.
